# Miscellaneous

**Published:** 1884-04

**Authors:** 


### Miscellaneous.

### BOOKS AND PAMPHLETS RECEIVED.

### BOOKS.

A Manual of Pathology. Coates. Philadelphia: Henry C. Lea's Son & Co. Jansen, McClnrg & Co.

Insanity in its Medico-Legal Relations. T. Buckham. Lippincott & Co. Jansen, McClurg & Co.

Venereal Diseases. Bumstead& Taylor. H. C. Lea's Son & Co Jansen, McClurg & Co.

Chemistry, Medical, General, &c. Attfield. Lea's Son & Co. Jansen, McClurg & Co.

Chemistry, Organic, Inorganic, &c. Bloxam. Fifth Edition. Lea's Son & Co. Jansen, McClurg & Co.

Manual of Practical Hygiene. Parkes. 2 Vols. Wm. Wood & Co. W. T. Keener.

Training Schools for Nurses. Notes on Twenty-two Schools. W. G. Thompson. Putnam's Sons. Jansen, McClurg & Co.

Hand-Book of Therapeutics. Ringer. Tenth edition. Wm. Wood & Co. W. T. Keener,

Materia Medica and Therapeutics. Bartholow. Fifth edition. W. T. Appleton & Co. Jansen, McClurg & Co.

Manual of Psychological Medicine. Edward C. Mann. Blakiston, Son & Co. W. T. Keener.

A Treatise on Syphilis in Children and Infants. P. Diday. Translated by Whitley. Wood & Co. W. T. Keener.

Medical Ethics and Medical Etiquette. A Symposium from the Liberal Standpoint.

Elements of Histology. Klein. H. C. Lea's Son & Co. Jansen, McClurg & Co.

Manual of General Medical Technology. Edw. Curtis. Wm. Wood & Co. W. T. Keener.

Transactions of the College of Physicians. Third series, Vol. VI. P. Blakiston.

Index to Transactions American Medical Association. Vols. I to XXXIII. W. B. Atkinson, Sec'y.

Parrish's Treatise on Pharmacy. Wiegand. H. C. Lea's Son & Co.

Legal Medicine. Tidy. Vol. II. H. C. Lea's Son & Co. Jansen, McClurg & Co.

St. Bartholomew's Hospital Reports. Vol. XIX., 1883. Smith, Elder, & Co., London.

Transactions American Surgical Association. Vol. I., 1881 to 1883. P. Blakiston, Son, & Co. W. T. Keener.

Surgical Diagnosis. Ranney. Third edition. Wm. Wood & Co. W. T. Keener.

Preventive Medicine. Richardson. H. C. Lea's Son & Co. Jansen, McClurg & Co.

Opera Minora. Seguin. G. P. Putnam's Sons. Jansen, McClurg & Co.

A System of Oral Surgery. Garretson. J. B. Lippincott & Co. S. A. Maxwell & Co.

Diseases of the Spinal Cord. Gowers. P. Blakiston, Son & Co.

Diseases and Injuries of the Horse. Kirby. Wm. Wood & Co. W. T. Keener.

Legal Medicine. Tidy. Vol. III. Wm. Wood & Co. Cheap series for 1884. W. T. Keener.

Applied Surgical Anatomy. Treves. H. C. Lea's Son & Co. Jansen, McClurg & Co.

Elements of Surgical Pathology. A. J. Pepper. H. C. Lea's Son & Co. Jansen, McClurg & Co.

Elements of Human Physiology. Power. H. C. Lea's Son & Co. Jansen, McClurg & Co.

Clinical Chemistry. Ralfe. H. C. Lea's Son & Co. Jansen, McClurg & Co.

The Dissector's Manual. Clarke. H. C. Lea's Son & Co. Jansen, McClurg & Co.

Influence of the Mind on the Body. Tuke. Second Am. from second Eng. edition. H. C. Lea's Son & Co. S. A. Maxwell & Co.

Voice, Song and Speech. Browne & Behnke. G. P. Putnam's Sons. Jansen, McClurg & Co.

Female Hygiene and Female Diseases. J. K. Shirk. Lancaster Publishing Co.

PAMPHLETS.

Transactions of the Massachusetts Medico-Legal Society. Vol. I., No. 6.

Concealed Insanity. (Case of Mark Gray). Brower.

Study of Neglected Lacerations of the Cervix Uteri. Ashby.

A Tracheotomy Tube for Gradual Withdrawal. Hendrix.

Antipyretic Treatment of Typhoid Fever. Smythe.

Alcoholic Insanity. Mason.

Treatment of the Pedicle in Ovariotomy. Sutton.

Treatment of Syphilis. Sims

The Cholera Scare. J. W. Reilly.

Human Parasites. Goding.

Serpiginous Phagedena. Scott.

The Mental Status of Guiteau. A Review. McBride.

Infusion of Jequirity in Inveterate Pannus. Peck.

Outline of the History, Theory, and Practice of Quarantine. Jones.

Medical Education in the United States and Canada. Illinois Board of Health.

Intestinal Obstruction and Operation. Parkes.

Laceration of the Cervix Uteri. Harvey.

Purulent Inflammation of the Middle Ear. Peck.

Diagnosis of Ovarian Tumors. Borck.

The Electro Osteotome. Roberts.

Training and Education of the Feeble-Minded and Imbecile. Davis.

General Paralysis of the Insane. McFarland.

Management of Enteric Fever. Wilson.

Delayed and Non-Union of Fractures. Senn.

Increase of Insanity in the United States. A Study of the Tenth Census. Pratt.

Address to the Medical Class at Dartmouth College. Elsberg.

Annual Report of the Surgeon-General U. S. A., 1883.

New Mode of Operating in Fistula in Ano. Jenks.

Report of the Section on Dental and Oral Surgery of the American Medical Association.

Jequirity in Granular Eyelids. Hotz.

Annual Report of the Librarian of the American Medical Association. Kleinschmidt.

Leprosy in Minnesota. Grönvold.

Arrest of Development from Uterine Pressure. Hendrix.

Study of a Case of Multiple Sarcoma of the Skin. Hyde.

Medical Symbolism. Sozinskey.

Perinephritic Abscess. Roberts..

